# Evolutionary history of histone demethylase families: distinct evolutionary patterns suggest functional divergence

**DOI:** 10.1186/1471-2148-8-294

**Published:** 2008-10-24

**Authors:** Xiaofan Zhou, Hong Ma

**Affiliations:** 1 The Intercollege Graduate Program in Cell and Developmental Biology, the Huck Institutes of the Life Sciences, the Pennsylvania State University, 415 Life Science Building, University Park, Pennsylvania 16802, USA; 2 Department of Biology, the Institute of Molecular Evolutionary Genetics, the Pennsylvania State University, 405D Life Science Building, University Park, Pennsylvania 16802, USA; 3 School of Life Sciences, the Institute of Plant Biology, the Center for Evolutionary Biology, the Institutes of Biomedical Sciences, Fudan University, Shanghai 200433, PR China

## Abstract

**Background:**

Histone methylation can dramatically affect chromatin structure and gene expression and was considered irreversible until recent discoveries of two families of histone demethylases, the KDM1 (previously LSD1) and JmjC domain-containing proteins. These two types of proteins have different functional domains and distinct substrate specificities. Although more and more KDM1 and JmjC proteins have been shown to have histone demethylase activity, our knowledge about their evolution history is limited.

**Results:**

We performed systematic phylogenetic analysis of these histone demethylase families and uncovered different evolutionary patterns. The *KDM1 *genes have been maintained with a stable low copy number in most organisms except for a few duplication events in flowering plants. In contrast, multiple genes for JmjC proteins with distinct domain architectures were present before the split of major eukaryotic groups, and experienced subsequent birth-and-death evolution. In addition, distinct evolutionary patterns can also be observed between animal and plant histone demethylases in both families. Furthermore, our results showed that some *JmjC *subfamilies contain only animal genes with specific demethylase activities, but do not have plant members.

**Conclusion:**

Our study improves the understanding about the evolutionary history of *KDM1 *and *JmjC *genes and provides valuable insights into their functions. Based on the phylogenetic relationship, we discussed possible histone demethylase activities for several plant JmjC proteins. Finally, we proposed that the observed differences in evolutionary pattern imply functional divergence between animal and plant histone demethylases.

## Background

One important mechanism for eukaryotic gene regulation is the epigenetic regulation of chromatin structure. The basic unit of chromatin is the nucleosome, which consists of 146 bp of DNA wrapped around an octamer of four histone proteins, H2A, H2B, H3, and H4. Histone proteins can be modified on the N-terminal tail and the modifications can disrupt the interaction between nucleosomes to prevent the packaging of chromatin into higher order structures; also the modified tails can serve as binding sites for chromatin modifiers, facilitating their functions [1]. Histone modifications, such as methylation and acetylation, have been well studied and many of the sites for the modifications are known [1]. For example, methylation can take place on several lysine residues on histone H3 and H4 (H3K4, H3K9, H3K27, H3K36, etc.) and each lysine residue can be mono-, di- or trimethylated. Histone arginine residues like H3R2 and H4R3 can also be mono- or dimethylated. According to the histone code hypothesis, different histone modifications are linked to distinct functional outcomes: H3K4 and H4K36 methylations are mainly associated with active genes while methylated H3K9 and H3K27 are markers for the repressed chromatin in general [1,2].

As important mechanisms of gene regulation, histone modifications themselves are under precise control [1]. It is known that many histone modifications are dynamically regulated by enzymes which add or remove the chromatin modifications, with defects in either of these two functions resulting in incorrect activation or repression [1]. However, histone methylation was considered irreversible for a long time. Although histone methylation was first reported in 1964 and the first histone methyltransferase was discovered in 2000 [3,4], it was not until 2004 that KDM1 [histone lysine (K) demethylase 1; previously known as LSD1 (Lysine specific demethylase)] was identified as the first histone demethylase [5]. KDM1 contains a C-terminal amine oxidase (AOD) domain, which is responsible for the demethylase activity through a flavin adenine dinucleotide (FAD)-dependent mechanism, and an N-terminal SWIRM domain also found in other chromatin regulators [5]. Several studies showed that the SWIRM domain is important for the stability and chromatin targeting of KDM1 [6-8]. Since the chemical mechanism of KDM1 mediated demethylation requires a protonated nitrogen for the reaction to proceed, the substrate specificity of KDM1 is limited to mono- or dimethylated lysine residues [9]. Types of histone methylation shown by biochemical studies to be demethylated by KDM1 include H3K4me1/2, and in the presence of androgen receptor (AR), H3K9me1/2, representing a small subset of all the possible states of histone methylation [10].

Soon after the identification of KDM1, the Jumonji C (JmjC) domain-containing proteins were discovered to be another family of histone demethylases [11]. The JmjC domain is the catalytic domain and these proteins belong to the Cupin superfamily of Fe(II) and α-ketoglutarate dependent dioxygenases [12]. Unlike KDM1, the JmjC domain-containing proteins that have been tested do not require a protonated nitrogen and are able to reverse all three states of lysine methylation [9]. Members in this family have been shown to be able to remove the methyl groups on H3K4, H3K9, H3K27 and H3K36 [13]. Furthermore, a protein in this family, the JMJD6, functions as a histone arginine demethylase through a similar chemical mechanism [14]. JmjC proteins usually contain additional domains, which are involved in the recognition of methylation (e.g. PHD and Tudor), protein-protein interaction (e.g. F-box) and DNA binding (e.g. C2H2 zinc finger), suggesting a wide range of possible functional interactions.

The number of studies of histone demethylases is increasing rapidly in recent years, with members in both families shown to have important biological functions. *KDM1 *is an essential gene in mouse [15] and important for viability and fertility in *Drosophila *[16]. The *Arabidopsis *homologs of *KDM1*, including *Flowering Locus D *(*FLD*), regulate the transition to reproductive development [17-20]. Moreover, the JmjC domain-containing proteins are involved in a broad range of processes. For example, the newly identified H3K27 demethylases, UTX and JMJD3, play important roles in regulating *Hox *gene expression and the animal body development [21,22]. In addition, JMJD3 was suggested to function in the neural stem cell differentiation [23]. Other JmjC domain-containing proteins are involved in processes such as the X-linked neural development (JARID1C) [24,25] and embryonic stem cell self-renewal (JHDM2A and JHDM3C) [26].

While these studies greatly advanced our understanding about the molecular and biological functions of histone demethylases, they only covered a limited fraction of the proteins in the two histone demethylase families. A large number of KDM1 and JmjC-containing proteins remain to be functionally characterized, especially in plants. There are only very few studies on plant histone demethylases. In addition to *FLD *and its two relatives, only three JmjC domain-containing proteins in *Arabidopsis *have reported functional studies [27,28], although it is reasonable to expect that the plant histone demethylases have important functions.

Phylogenetic analyses can provide useful information about evolutionary relationship among related genes from different organisms and clues about possible functions of genes closely related to those with known functions. Furthermore, the differences in evolutionary pattern between gene families or species also suggest different evolutionary pressures and diverged functions. Homologs of both types of histone demethylase have been detected in major groups of eukaryotes [5,12]. However, to our knowledge, there is no detailed phylogenetic analysis on the KDM1 proteins and only one report exists for JmjC domain-containing proteins from fungi and animals [29]. To gain a better understanding of the evolutionary history of these two histone demethylase families, we performed systematic phylogenetic analyses in this study including sequences from eukaryotes and bacteria. We also discussed the functional implications of the evolutionary patterns we observed in the two families.

## Results and discussion

### Distribution of AOD domain-containing proteins in major lineages

Since the AOD domain is the catalytic domain in the KDM1-type histone demethylases, we collected gene sequences for AOD domain proteins from selected animal, plant and fungal species following the procedure described in Methods. In total, 118 sequences were retrieved from 12 organisms (Table 1, Additional file 1). The *AOD *genes are present in Eukaryotes and Eubacteria, but absent in Archaea. In this study, all *AOD *genes were named based on their domain structure. The genes which encode proteins with only the AOD domain were named as *AOD *genes, whereas the genes coding for proteins with both the SWIRM and the AOD domain were named as *KDM1*. *KDM1 *genes only exist in Eukaryotes, and account for only a small fraction of the *AOD *genes (e.g., 2/8 in human and 4/14 in *Arabidopsis*). The *KDM1 *genes have maintained a constant copy number of two in most animal species from the basal invertebrate sea anemone to human, except for insects and several nematodes, which contain one and three copies, respectively. A different trend was observed in plants. The number of *KDM1 *genes increased from 2 in green algae to 4 in *Arabidopsis *and rice, with the highest number of 7 in poplar (*P. trichocarpa*), which is thought to have experienced a relatively recent genome-wide duplication [30].

**Table 1:** Number of AOD domain-containing genes and JmjC domain-containing genes included in this study:

Organism	AOD domain-containing gene		JmjC domain-containing gene
		
	*KDM1*	*AOD*	
Human	2	6	26
Zebrafish^b^	2	5	
*Drosophila*	1	7	12
*C. elegans*	3	4	12
Mouse^a^	2		
Pufferfish^a^	2		
Sea squirt^a^	2		
Sea urchin^a^	2		
Mosquito^a^	1		
Honey bee^a^	1		
Beetle^a^	1		
Sea anemone^a^	2		
*Arabidopsis*	4	10	19
Poplar	7	17	25
Rice	4	10	15
*Selaginella*^b^	2	10	
Moss	3	10	14
*Ostreococcus*^b^	2	6	
Fission yeast	2	0	5
Budding yeast	0	1	4

a. Only *KDM1 *genes are collected in these organisms.

b. Only *AOD *genes are collected in these organisms

In fungi, *KDM1 *was found in the fission yeast *Schizosaccharomyces pombe *but not the budding yeast *Saccharomyces cerevisiae *[5]. To investigate the distribution of *KDM1 *in fungi, we searched for *KDM1 *genes in completely sequenced fungal genomes in the NCBI database. Our phylogenetic analysis of the fungal *KDM1 *sequences (see Additional file 2) indicates that one *KDM1 *gene was present in the ancestor of Ascomycota and it was lost in the common ancestor of the budding yeast and *Candida albicans *after its divergence from *Y. lipolytica*. In fission yeast, the two *KDM1 *genes were shown to have important functions in regulating heterochromatin [31,32], which is marked by H3K9 methylation. In contrast, the budding yeast does not possess H3K9 methylation and employs a different set of proteins to fulfill the function of fission yeast *KDM1 *genes in heterochromatin regulation [33]. Furthermore, in the absence of *KDM1 *homolog, The H3K4 demethylation in the budding yeast is performed by a JmjC domain protein which will be discussed later.

### Phylogenetic analyses of *AOD *genes

**Figure 1:**
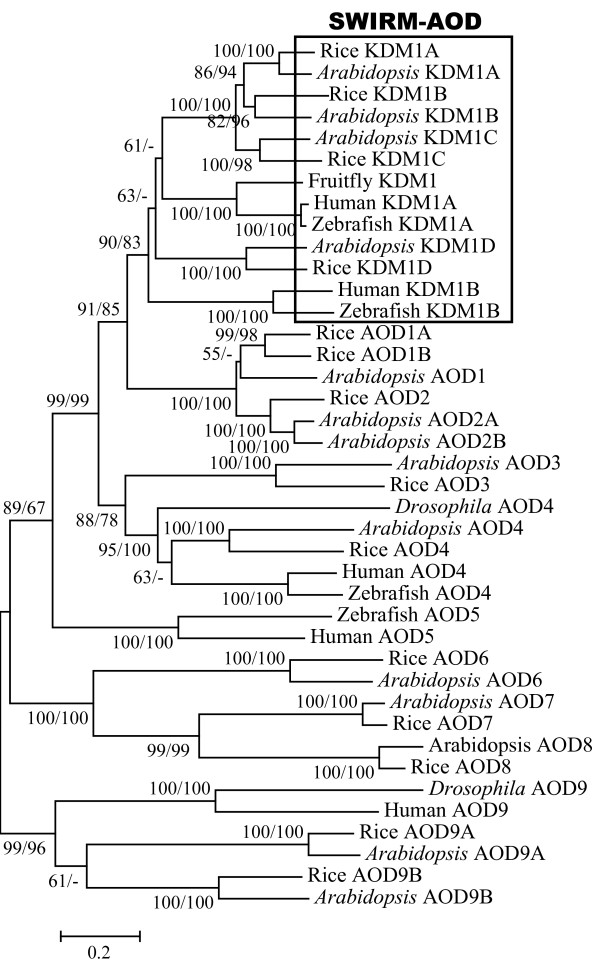
**Phylogenetic tree of *AOD *genes from representative plant and animal species using the AOD domain region.** Both NJ and ML methods were used to infer the evolutionary history, and only the NJ tree is shown. NJ/ML bootstrap values are presented for clades with support greater than 50%. All *KDM1 *genes which code for proteins with both SWIRM and AOD domains form a single clade, which is highlighted in the box. The remaining *AOD *genes form six well supported clades.

To investigate the evolutionary history of *AOD *genes, we carried out phylogenetic analyses with sequences from representative species using both NJ and ML methods, yielding very similar results. The phylogenetic tree (Fig. 1) indicates that all *KDM1 *genes form a single clade with 90/83 bootstrap support. Within this clade, the animal *KDM1 *genes form two highly supported (100/100) groups, each contains one *KDM1 *gene from the two vertebrates, human and zebrafish. The only *Drosophila KDM1 *gene is in the same group as the human *KDM1A *gene. Similarly, the plant *KDM1 *genes are also divided into two separate groups, each with 100/100 support. The relationship between these animal and plant groups is unclear since the topology lacks strong bootstrap support. However, our results still suggest an early origin of *KDM1 *genes prior to the divergence of animals and plants. Besides the *KDM1 *clade, there are six major clades of *AOD *genes. One of these clades contains both animal and plant *AOD *genes, three are plant specific and one is animal specific. Based on these results, it could be estimated that, in the most recent common ancestor of animals and plants, there were at least one *KDM1 *gene and six additional *AOD *genes.

Previous studies showed that the human KDM1A protein has an insertion in the AOD domain [5]. The insertion forms a coiled-coil protruding from the AOD domain and is required for the binding between human KDM1 and CoREST [6,8,34]. The alignment of AOD amino acid sequences showed that this insertion is conserved among animal KDM1A. The fungal KDM1 proteins also have an insertion at the same position, but the sequences are not similar to the animal insertions. Insertions of much shorter length can also be detected in several plant KDM1 proteins. By contrast, no insertion was found in other AOD proteins.

We used the COILS program to test whether the insertions in different KDM1 proteins are able to form a coiled-coil structure. Consistent with the crystal structure, the insertions in the human KDM1A protein is predicted to form a coiled-coil structure with high support. The same results were obtained for other animal KDM1A proteins, suggesting that the interaction between human KDM1A and CoREST might be conserved in all animals. The lack of insertion in animal KDM1B suggests a functional divergence between these two proteins. Interestingly, although the fungal KDM1 proteins possess an insertion, no coiled-coil is predicted. Several studies showed that the two *S. pombe *KDM1 proteins form a complex with two PHD domain-containing proteins [35]. Hence, unlike their counterparts in animal, the insertions in fungal KDM1s might be involved in the interaction with these PHD proteins or have other functions. The absence of the insertion in other KDM1 proteins suggests that this insertion is not essential for the histone demethylase activity. Alternatively, the KDM1 proteins without the insertion might have different activities.

### Plant and animal *KDM1 *genes have different evolutionary patterns

**Figure 2:**
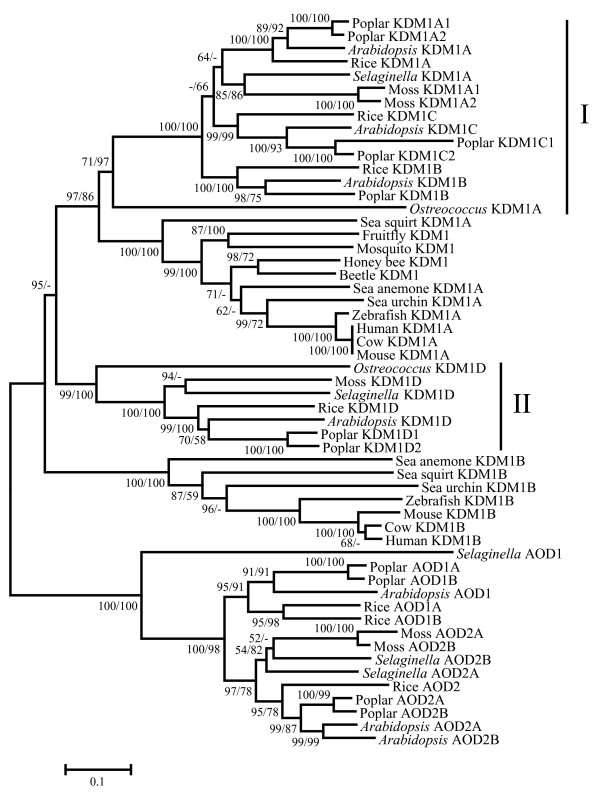
**Phylogenetic tree for *KDM1 *genes with *AOD1 *and *AOD2 *genes as outgroup. **The tree was constructed using the AOD domain region. Plant and animal *KDM1 *genes each form two separate clades; the plant specific group I and group II, and the animal specific KDM1A and KDM1B. The methods for tree construction and bootstrap values are given as in Fig. 1.

To further understand the evolution of *KDM1 *genes in different lineages, *KDM1 *genes from more species were included in the phylogenetic analysis. A representative phylogenetic tree shown in Fig. 2 has high bootstrap supports for the two animal clades and two plant clades of *KDM1 *genes. In this tree, the plant group I and animal *KDM1A *group cluster together to form a clade with 97/86 bootstrap support. The plant group II is placed outside this clade, and the animal *KDM1B *group occupies the basalmost position in the *KDM1 *clade. However, while the position of plant group II is highly supported (95) in the NJ tree, it has no support from the ML method. This discrepancy between the bootstrap values from two methods might be due to the long branches of the animal *KDM1B *genes. Therefore, according to these results, there were at least two copies of *KDM1 *genes present in the most recent common ancestor of animals and plants. Furthermore, the inclusion of additional sequences revealed distinct evolutionary patterns of animal and plant *KDM1 *genes. The animal *KDM1A *and *KDM1B *genes both maintain only one copy in most animals. However, *KDM1B *was not found in insects, implying that it was lost in the ancestor of insects. By contrast, the plant group I contains three subgroups and each subgroup consists of genes from monocots and eudicots, indicating the presence of three copies of group I *KDM1 *genes in the most recent common ancestor of angiosperms. Due to the lack of complete genomic sequence and EST data, we did not detect sequences from gymnosperms. Hence it is unclear how many *KDM1 *genes were present in the ancestor of seed plants. However, the basalmost position of the green algae *KDM1A *in this group suggests that all members in group I were derived from a single copy of *KDM1 *in the ancestor of green plants. In addition, lineage specific duplication events were found in moss and poplar, as well as in group II.

Several genome-wide studies in fungi and *Drosophila *suggested that evolutionary patterns of gene families are correlated to their functions [36,37]. The genes with low volatility in copy number during evolution are usually associated with essential functions. In fact, the *KDM1A *genes have been shown to be essential in mouse and *S. pombe *and are involved in important biological processes like meiotic progression and spermatogenesis [31,38]. Although the function of animal *KDM1B *genes is not known, the similarity of their evolutionary pattern to that of *KDM1A *also implies functional conservation and importance. Consistent with this idea, the residues critical for cofactor binding and catalytic activity are conserved in animal KDM1B proteins, suggesting that they have histone demethylase activity. Moreover, the expression of animal *KDM1B *gene is supported by considerable amount of EST data, although less abundant than *KDM1A*.

However, several potential substrate-binding residues are substituted in animal KDM1B, suggesting possible changes in substrate specificity of these proteins. Other lines of evidence also support the functional divergence between animal *KDM1A *and *KDM1B *genes. Besides the SWIRM and the AOD domain, the animal KDM1B proteins also contain a CW-type zinc finger near the N-terminus. The function of this zinc finger is not well characterized, but it is usually found in proteins which also have other domains involved in DNA binding or protein-protein interaction [39]. Interestingly, this domain is also found in a class of SET domain histone methyltransferases (HMTs), which have H3K36 methyltransferase activity [40]. Therefore, the zinc finger possibly facilitates the recognition of substrates other than methylated H3K4 and H3K9 by KDM1B. Furthermore, the tree in Fig. 2 also shows that the animal *KDM1B *genes have branches longer than those of *KDM1A*, indicating that the *KDM1B *genes have evolved at higher rates. To test this idea, we also conducted Ka/Ks analyses for several pairs of animal genes. The results indicate that: (1) both *KDM1A *and *KDM1B *genes were under purifying selection with Ka/Ks ratio lower than 0.1; (2) Ka/Ks values for *KDM1B *genes were significantly higher than those for *KDM1A *genes, indicating that the *KDM1B *genes have evolved under less stringent selective pressure (see Additional file 3). As all these results point to a functional divergence between animal *KDM1A *and *KDM1B*, it will be worth investigating the functions of KDM1B proteins in the future.

In plants, our results showed that the copy number of group I *KDM1 *gene increased from one in the common ancestor of green plants to three in the common ancestor of flowering plants. The functional studies of *AtKDM1A*, *AtKDM1B *and *AtKDM1C *revealed that all three genes regulate the transition to reproductive development [17,18]. It is possible that the expansion of plant group I might have contributed to the evolutionary success of flowering plants. The initiation of reproductive development is one of the most important developmental events in plants and is regulated by a complex regulatory network [41]. According to the duplication-degeneration-complementation (DDC) model [42], the duplicate group I *KDM1 *genes would have undergone sub-functionalization or neo-functionalization, which might help to optimize the regulatory network controlling flowering. In fact, functional studies showed that these three genes have partially redundant functions in the repression of the expression of *FLC*, a major inhibitor of flowering [17,18]. In addition, *AtKDM1B *and *AtKDM1C *can also affect the expression of *FWA*, a function independent of that of *AtKDM1A*.

In contrast, such duplication events were not observed for group II genes, suggesting a difference in the function between group I and group II *KDM1 *genes. Since there is no reported study on *AtKDM1D*, it is unclear whether this gene also participates in the regulation of flowering. The expression data from the GENEVESTIGATOR database and our previous microarray results [43,44] showed that *AtKDM1D *is expressed at very low levels across all developmental stages. On the other hand, the sequence of *AtKDM1D *gene is well conserved. Hence it is possible that *AtKDM1D *has evolved a function in a specific group of cells or for a specific environmental situation.

### The origin of SWIRM-AOD architecture

**Figure 3:**
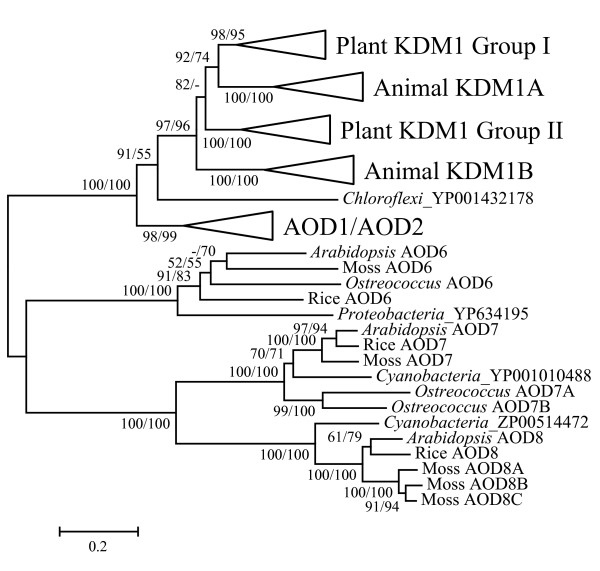
**Phylogenetic tree showing the possible origin of selected eukaryotic *AOD *genes.** The tree includes *KDM1*, *AOD6*, *AOD7 *and *AOD8 *genes and their most closely related eubacterial homologs. The tree was constructed using AOD domain region. The methods for tree construction and bootstrap values are given as in Fig. 1.

To investigate the origin of the *KDM1 *genes, we performed additional phylogenetic analysis with eukaryotic *AOD *genes and the most similar *AOD *genes from Eubacteria. Our results (Fig. 3) showed that most major clades have one eubacterial *AOD *gene at or near the basal position. The *R. castenholzii AOD *gene is placed at the basal position outside all *KDM1 *genes with 91/55 bootstrap support values. This topology suggests that all *KDM1 *genes have a single origin from an *AOD *gene in the ancestor of Eukaryotes and Prokaryotes. However, it is still not clear whether the plant *AOD1 *and *AOD2 *genes have the same origin as *KDM1 *since the position of *R. castenholzii AOD *gene was only weakly supported by the ML method.

As the KDM1 proteins also contain a SWIRM domain in addition to the AOD domain, how the SWIRM-AOD domain architecture originated is still a question. According to previously proposed mechanisms for the evolution of new gene structures [45], there might be two possible origins for the first *KDM1 *gene: (1) an exon shuffling/retrotransposition event that brought these two domain together; (2) *de novo *evolution of SWIRM domain coding region at the 5' of a preexisting *AOD *gene. Previous studies have shown that, in spite of its short length, the SWIRM domain is an evolutionarily conserved domain that occurred in proteins with different domain compositions [46]. Therefore the second possibility is unlikely.

**Figure 4:**
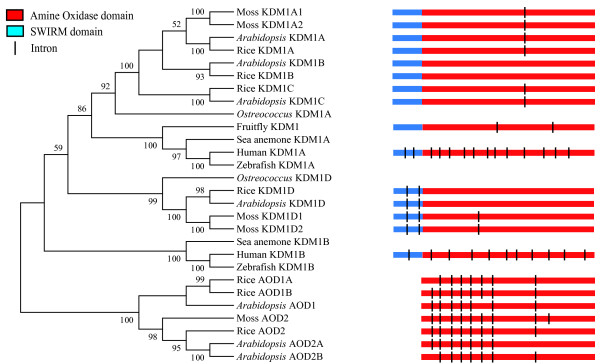
**Schematic diagram of intron-exon structure of *KDM1*, *AOD1 *and *AOD2 *genes.** Only the introns in SWIRM and AOD domain coding region are shown. The exons are drawn to scale.

To explore the first possibility, we analyzed the intron/exon structures of the *KDM1 *genes and the closely related *AOD *genes (Fig. 4). Among the plant *AOD1 *and *AOD2 *genes, the number of introns ranged from 7 to 9 in the AOD domain-coding region. With the exception of only a few intron loss and gain events, the positions of all the introns are highly conserved. In contrast, the plant *KDM1 *genes have many fewer introns. All plant *KDM1D *genes have two introns in the SWIRM domain-coding region and, except for the two *PpKDM1D *genes, have no intron in the AOD domain. Interestingly, the other plant *KDM1 *genes have no intron in the SWIRM domain, but most of them possess an intron in the AOD domain at a different position from all the other introns mentioned above, and the *AtKDM3B *and *OsKDM3B *are intronless for the entire gene. These intron/exon structures are also conserved in poplar and grape (*V. vinifera*) (not shown) [30,47].

In comparison to the few introns observed in plant *KDM1 *genes, the animal *KDM1 *genes exhibit completely different patterns of intron positions. Most of the animal *KDM1 *genes have one or two introns in the SWIRM domain and around 10 introns in the AOD domain, with the exception of insect *KDM1 *genes, which have only 2 introns in the AOD domain only. Furthermore, although the positions of introns are conserved among animal *KDM1A *and *KDM1B *genes respectively, they are different from each other or that of the plant *KDM1 *genes.

The most parsimonious explanation for the observed intron patterns is that the AOD domain of the ancestral *KDM1 *gene in the most recent common ancestor of animals and plants was intronless, which supports the origin of *KDM1 *gene through retrotransposition. After that, the plant *KDM1 *genes have experienced limited or no intron gains, whereas the animal *KDM1 *genes accumulated many introns during their evolution. It is still not clear what evolutionary pressure suppressed intron gain in plant *KDM1 *genes. Nevertheless, these results again clearly support our conclusion that the animal and plant *KDM1 *genes experienced very different evolutionary history.

### Evidence for horizontal gene transfer (HGT) during the evolution of *AOD *genes

**Figure 5:**
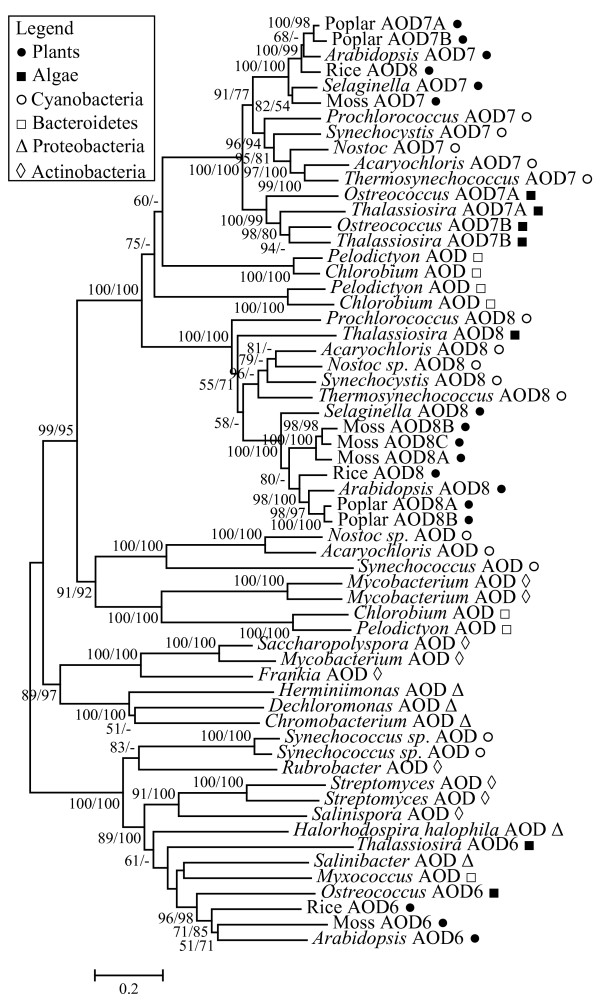
**Phylogenetic tree of eukaryotic *AOD6*, *AOD7 *and *AOD8 *genes and their homologs from selected bacterial species, showing possible horizontal gene transfer between bacteria and plants.** The tree was constructed using AOD domain region. The methods for tree construction and bootstrap values are given as in Fig. 1.

Another interesting result worth noting in Fig. 3 is that the most closely related eubacterial homologs of the plant *AOD7 *and *AOD8 *genes, respectively, are both from cyanobacteria. Previous studies revealed that both AtAOD7 and AtAOD8 proteins have chloroplast targeting signal and are localized to chloroplast [48]. It has been proposed that chloroplast originated from an eubacterium related to cyanobacteria through an endosymbiotic event, after which many genes have been transferred from the chloroplast to the nuclear genome [49]. Hence it is highly possible that the plant *AOD7 *and *AOD8 *genes are derived from the chloroplast. To examine this possibility, we performed further phylogenetic analysis that included the plant *AOD7 *and *AOD8 *genes and their eubacterial homologs. Besides plants, the only eukaryotic species in which we were able to find homologs of *AOD7 *and *AOD8 *was the brown alga *T. pseudonana*, which was proposed to have acquired a chloroplast through a secondary endosymbiotic event [50]. As shown in Fig. 5, the eukaryotic *AOD7 *and *AOD8 *genes cluster with their respective homologs from cyanobacteria with high bootstrap support (100/100). These results together suggest a cyanobacterium-like origin of the eukaryotic *AOD7 *and *AOD8 *genes.

AOD7 and AOD8 both are key enzymes important for the biosynthesis of carotenoids in all photosynthetic organisms, including plants, algae and cyanobacteria [51]. In the carotenoid synthetic pathway, these two enzymes catalyze the two dehydrogenation reactions that convert phytonen to *cis*-lycopene, which is then converted to all-*trans*-lycopene by an isomerase [48]. In many nonphotosynthetic organisms like fungi and eubacteria except cyanobacteria, these three steps are replaced by a single reaction that is catalyzed by a distinct enzyme [48]. It is possible that these two *AOD *genes were recruited to the carotenoid pathway in the common ancestor of cyanobacteria after its divergence from the other eubacteria, and then the photosynthetic eukaryotes acquired these two genes from cyanobacteria through HGT.

The plant-specific *AOD6 *group, which is closely related to *AOD7 *and *AOD8 *groups, also shows a similar pattern. A homolog of plant *AOD6 *genes can be found in the brown alga *T. pseudonana*, but not in animals and fungi. The AOD6 proteins are predicted to have a chloroplast-targeting signal, but the actual localization and function remains unknown. In addition, the region of the *AOD6 *genes encoding the AOD domain is intronless. Our phylogenetic analysis (Fig. 5) showed that eukaryotic *AOD6 *genes are most closely related to *AOD *genes from proteobacteria and bacteroidetes, suggesting that the eukaryotic *AOD6 *genes also originated through HGT from a eubacterium. The results on *AOD6*, *AOD7*, and *AOD8 *phylogeny together reveal an important role of HGT events in the evolution of *AOD *genes.

### JmjC domain-containing proteins

Klose *et al. *have studied the evolutionary relationship between animal and fungal JmjC domain-containing proteins and they identified seven subfamilies based on both phylogenetic analysis of JmjC domain and domain architecture information [29]. JmjC proteins in six of the seven subfamilies have multiple domains and each family has a distinct domain structure. However, the evolutionary history of plant JmjC proteins is not clear. It has already been reported that two *Arabidopsis *JmjC proteins have an unusual domain architecture, which is not found in animals and fungi. Hence it will be of interest to elucidate the phylogeny of plant JmjC proteins, and compare between the evolutionary patterns of plant and animal JmjC proteins. To investigate the evolutionary history of plant JmjC domain histone demethylase genes, we retrieved sequences for JmjC domain-containing proteins from various plants and selected animals (Table 1, Additional file 1). We also used the sequences of eukaryotic JmjC domains as queries to search for JmjC domain-containing proteins in prokaryotes. While proteins with limited similarity were found in Eubacteria, no homolog was detected in Archaea. Thus, our results indicate that neither AOD nor JmjC protein is present in Archaea. It is known that some archaea already possess the pseudonucleosomal tetrameric structures [52]. However, the absence of histone demethylase in Archaea is not surprising since archaeal histone proteins do not have N-terminal tails [52]. It is possible that, upon the acquisition of histone tails in early eukaryotes, AOD and JmjC proteins were recruited to serve as chromatin modifying enzymes.

The JmjC domains in most eubacterial JmjC proteins have low support from the SMART analysis and they are annotated as Cupin domain by Pfam with high e-value. Similarly, the JmjC domains in human MINA53, NO66, *Drosophila *CG2982 proteins retrieved in this study are also annotated as Cupin with strong support. The domain architecture analysis of the collected proteins shows that all the eubacterial proteins have only the JmjC domain. In contrast, most eukaryotic proteins contain other domain(s) besides the JmjC domain. Some domain architectures were observed only in plant members and others only in animals and/or fungi members. From the amino acid sequence alignment, the proteins with the same domain architecture have more similar JmjC domains and regions flanking the JmjC domains.

### The birth-and-death evolution of genes encoding JmjC domain proteins

**Figure 6:**
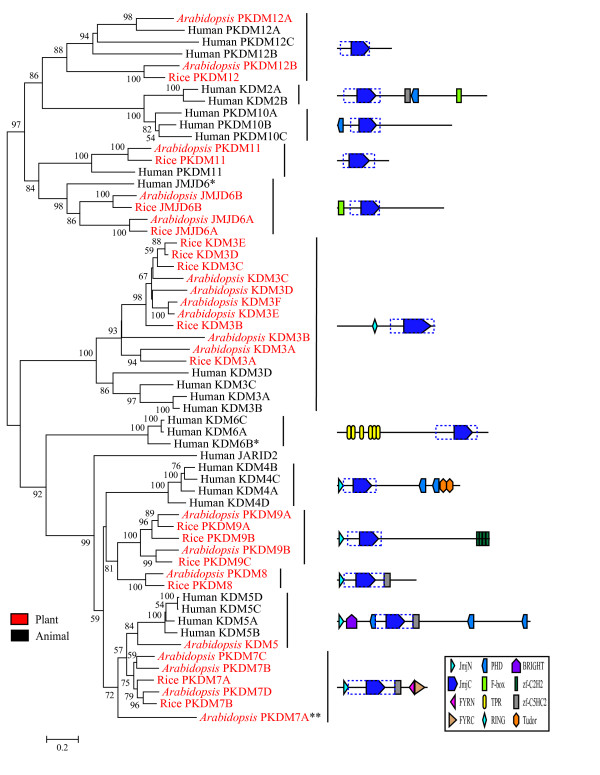
**NJ tree showing the evolutionary relationship between *JmjC *genes from Human, *Arabidopsis *and Rice.** The tree was constructed using JmjC domain region. Based on both phylogenetic information and domain architecture, 12 subfamilies can be defined. The representative domain architectures of each subfamily are shown next to the tree. JmjC proteins in the same subfamily have conserved regions extend from boundaries of JmjC domain, which are highlighted by dash line box. *: Human JMJD6 and KDM6B do not have additional domain besides JmjC domain. **: *Arabidopsis *PKDM7A has similar domain architecture to other PKDM7 proteins, but lacks the FYRN and FYRC domains. It is assigned to PKDM7 subfamily based on the phylogenetic tree shown in Fig. 7A.

According to the NJ tree shown in Fig. 6, the *JmjC *family can be divided into 12 monophyletic subfamilies. These 12 subfamilies represent 11 different domain architectures, as two subfamilies contain only the JmjC domain. Previously, these two subfamilies were in a monophyletic group with low support and was defined as a single subfamily [29], but our study supports two separate subfamilies. On the other hand, the other six subfamilies defined in the previous study [29] were confirmed by our result. Most of the 12 subfamilies are designated after the name of their animal members according to their chromatin modifying enzyme activities [53]. Among these subfamilies, the *KDM2*, *KDM4 *and *KDM6 *subfamilies are animal specific, while *KDM3*, *KDM5 *and *JMJD6 *have members from both plants and animals. Those subfamilies without a known histone demethylase function are named as PKDM (**P**utative-**KDM**). Among these, the *PKDM7*, *PKDM8 *and *PKDM9 *subfamilies are composed of only plant genes and *PKDM10 *is animal specific; the remaining two subfamilies, *PKDM11 *and *PKDM12*, contain both plant and animal genes. According to the tree topology, it could be estimated that there were at least nine JmjC genes in the most recent common ancestor of plants and animals. After the divergence of animals and plants, some copies were lost in plants, others in animals. When the human *MINA53*, *NO66*, *Drosophila CG2982 *genes and their eubacterial homologs encoding Cupin proteins were included in the analysis, they form a separate clade that is sister to the *PKDM12 *subfamily. Therefore it is possible that all *JmjC *genes originated from the ancestor of these *Cupin *genes.

**Figure 7:**
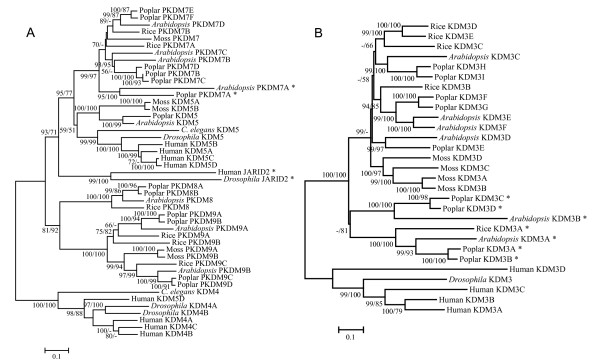
**Phylogenetic trees of *JmjC *subfamilies.** (A) A phylogenetic tree of JmjC genes in *KDM4*, *KDM5*, *PKDM7*, *PKDM8 *and *KDM9 *subfamilies. The tree was constructed using JmjN and JmjC domain regions as well as another region which is conserved among these five subfamilies. (B) A phylogenetic tree of *KDM3 *subfamily. The tree was constructed based on RING domain, JmjC domain and other conserved regions inside this subfamily. Proteins with mutated cofactor binding sites are indicated by asterisk.

Besides the above mentioned loss of specific subfamilies in plant or animal *JmjC *genes, three different patterns of birth-and-death evolution were also observed within the subfamilies. In the *PKDM9 *and *PKDM11 *subfamilies, a single copy has been stably maintained in both animals and plants, except for a recent duplication of *PKDM9 *in poplar. In other subfamilies, *JmjC *genes experienced duplication in one of the animal and plant lineages, but were stable or lost in the other lineage. For example, in the *JMJD6 *subfamily, while one copy has been maintained in each animal, two gene duplication events can be detected in plants, one before the divergence of land plants and another in moss. In contrast, the *KDM5 *subfamily has four members in humans, resulting from duplication events after the divergence of vertebrate animals from insects. This pattern is also found in the plant (e.g. *PKDM3*) or animal (e.g. *KDM6*) specific subfamilies. A third pattern is that *JmjC *genes were duplicated in both animals and plants, such as the *KDM3 *subfamily. As shown in Fig. 7A, five well supported clades all include members from *Arabidopsis *and poplar, suggesting the presence of five *KDM3 *genes in the most recent ancestor of *Arabidopsis *and poplar. These five clades were all derived from possibly one copy in the ancestor of plants and animals through gene duplication. In addition, lineage specific gene duplication events can be observed in plants. In animals, duplication events can also be inferred from the clade with 100/100 supports that is composed of one *Drosophila KDM3 *and four human *KDM3 *genes.

### Potential histone demethylase activities of plant JmjC proteins

Among the twelve subfamilies identified in this study, six of them have at least one member with known histone demethylase activities [13]. However, as all functional studies so far are performed in animals and fungi, no plant JmjC protein in these six subfamilies has been reported to have histone demethylase activity. In the absence of biochemical studies, our phylogenetic results can be valuable clues about possible functions of plant JmjC proteins. Here, we propose potential histone demethylase activities for the plant JmjC proteins based on the evolutionary relationships from this study, the conservation of enzymatic active sites and domain architectures.

As described above, three subfamilies have both members with known biochemical activities and members from plants. Two human proteins in *KDM3 *subfamily, human KDM3A and KDM3B, have been shown to have H3K9me1/2 demethylase activity [54]. The plant KDM3 proteins have the same domain architecture as the animal members, with a zinc finger domain in addition to the JmjC domain. The predicted cofactor binding sites are also conserved in most plant KDM3 proteins, suggesting possible H3K9 demethylase function. Consistent with this idea, a recent study revealed an increased level of H3K9 methylation at the *BNS *locus in the *Arabidopsis kdm3c *mutant [28]. However, proteins in the two clades including *Arabidopsis *KDM3A and KDM3B have variant residues at the co-factor binding sites. Hence it is possible that they have evolved novel functions or become pseudogenes. To investigate these two possibilities, we examined the expression data of *Arabidopsis KDM3 *genes from our previous microarray analysis [44] and the GENEVESTIGATOR database [43]. *AtKDM3A *has the highest expression level among these genes at all the developmental stages, and *AtKDM3B *is also expressed, suggesting they are functional.

Similar phenomenon can be observed in the *PKDM7 *subfamily. All proteins in this subfamily are from plant and they contain a JmjN domain, a C5H2-zinc finger domain and C-terminal FYRN and FYRC domains. The cofactor binding sites are conserved in all members but the *Arabidopsis *and poplar PKDM7A proteins, which have evolved much faster than the other members. Nevertheless, the expression data shows that *AtPKDM7A *is expressed at a level comparable to *AtPKDM7B *and *AtPKDM7D *[43,44]. Although the AtPKDM7C protein has intact cofactor binding sites, it has no detectable expression. The phylogeny in Fig. 7B shows that the *PKDM7 *subfamily forms a clade with 99/97 bootstrap supports and is most closely related to the *KDM5 *subfamily. Several animal KDM5 proteins have been shown to have H3K4me2/3 demethylase activities [24,55-57]. Therefore, although the plant KDM5 and PKDM7 proteins have distinct domain architecture, they might have H3K4 demethylase activities.

Recently, the human JMJD6 protein was shown to have histone arginine demethylase activity [14]. Although most of the cofactor binding sites are conserved in plant JMJD6 proteins, the first KG binding site has been substituted by Ser and Ala in plant JMJD6A and JMJD6B proteins, respectively. It is unclear whether these substitutions will compromise the histone arginine demethylase activity of plant JMJD6 proteins. We noticed that *AtJMJD6A *and *AtJMJD6B *are expressed at a high level at specific developmental stages [43,44], suggesting that these proteins are functional.

In summary, we have used our phylogenetic results to propose histone demethylase activities for plant JmjC proteins in four subfamilies. The other plant JmjC proteins are either in the plant specific subfamilies *PKDM8 *and *PKDM9 *or in the subfamilies *PKDM11 *and *PKDM12 *which do not have an animal member with known biochemical activities. Nevertheless, some of these plant JmjC proteins have already been implicated in chromatin modification. For example, an elevated histone H4 acetylation level is observed at the *FLC *locus in the *Arabidopsis pkdm9a *mutant, which is phenotypically similar to the *kdm1a *mutant [27]. Moreover, it is still not clear which proteins are responsible for the H3K9me3, H3K27 and H3K36 demethylation in plants, since the *KDM2*, *KDM4 *and *KDM6 *subfamilies do not have a plant member. One possibility is that these demethylase activities in plants are carried out by some of the other JmjC proteins without a known function.

### Functional implications of differences in evolutionary patterns

Our phylogenetic analyses of these two histone demethylase families revealed a significant difference in evolutionary pattern between animal and plant proteins in both families. In the *AOD *family, the plant group I *KDM1 *genes were duplicated several times before the diversification of flowering plants and further in specific lineages, whereas the animal *KDM1 *genes have been maintained with a constant copy number in most species. The animal and plant JmjC domain-containing proteins show similar patterns of evolution in some subfamilies but not in the others. Furthermore, certain types of histone demethylation might be conducted by plant JmjC proteins in subfamilies different from the animal JmjC proteins. These results indicate a divergence in the regulation of histone methylation between animals and plants, consistent with the proposed divergent roles of histone methylation in different organisms [58]. In animals, both H3K9me2 and H3K9me3 are enriched in heterochromatin. However, in *Arabidopsis*, while the H3K9me2 is considered as a hallmark of heterochromatin, H3K9me3 is mainly found in euchromatin [58]. In animals, H3K9me3 demethylation is catalyzed by members of the *KDM4 *subfamily [59-62], which lacks plant members, suggesting that H3K9me3 demethylation in plants is catalyzed by proteins from another subfamily. Furthermore, previous phylogenetic analysis also revealed a similar evolutionary pattern in the HDAC families; one of the three major classes of SIR2 family of HDACs has members from animal but not plant, whereas the HD2 family is plant specific [63]. Thus, the functional and regulatory diversification might be a common feature of chromatin modification genes.

In addition, our study also showed distinct evolutionary patterns between the *AOD *and the *JmjC *families. Whereas the *KDM1 *genes only experienced limited duplication events and maintained relatively constant domain architecture in their history, the *JmjC *gene have evolved several types of domain architectures before the divergence of major eukaryotic groups and underwent further duplication subsequently. As suggested by genome-wide studies in *Drosophila *and fungi, such divergence in evolutionary patterns may indicate differences in functional essentiality [36,37]. The KDM1 histone demethylases are reported to have a variety of functions. In animal, KDM1 is required for the ligand-dependent transcriptional activation by nuclear hormone receptors [10,64]. It also plays important roles in cell differentiation, cell cycle control and spermatogenesis [38]. In addition, most of these functions are also shared by JmjC proteins. For example, members of the *KDM3 *and *KDM4 *subfamilies, which possess H3K9 demethylase activities, are also required for the steroid hormone induced gene expression [26,54,64] and KDM3A is crucial for spermatogenesis [65]. In addition, the KDM5A, an H3K4me2/me3 demethylase, has overlapping roles with KDM1 in the regulation of cell differentiation [55]. It is also possible that KDM1 has some distinct function from those of JmjC proteins. In fact, the work by Lan *et al. *showed that KDM1 is retained on the unmethylated H3K4 after its action and suggested a role of KDM1 in the prevention of H3K4 methylation [66].

Another explanation for the observed evolutionary patterns is that they reflect the difference in evolutionary potential of these two families of histone demethylase. Consistent with this idea, several lines of evidence support a greater functional potential of JmjC proteins than KDM1. First, the JmjC proteins have broader substrate specificity than KDM1 proteins. The requirement of a protonated nitrogen in KDM1-mediated demethylation limits the substrate specificity of KDM1 to mono- and dimethylated lysine residues. By contrast, JmjC proteins are able to demethylate all the three states of lysine methylation. In addition, KDM1 proteins are only known to catalyze the demethylation on H3K4 and H3K9, whereas the substrates for JmjC proteins include H3K4, H3K9, H3K27, H3K36 and even H3R2. Studies on protein structures suggest that the interactions between KDM1 and the substrate are intricate and specific, leading to the exquisite substrate specificity of KDM1. Second, the JmjC domain is much smaller than the AOD domain in KDM1. The JmjC domain in most JmjC proteins are less than 200 amino acids, but the length of the AOD domain is usually more than 400 amino acids. Smaller domain might be combined with the other domains more easily, providing JmjC proteins greater evolutionary adaptability. This is supported by a recent study, which identified the protein domains with relatively high tendency to combine with different domains in eukaryotes [67]. In their list of highly versatile domains, most have 250 or fewer amino acids residues. Hence the short length of JmjC domain may allow JmjC proteins to evolve new functions quickly by combining with new domains, which can promote protein-protein interaction, DNA binding or recognition of chromatin modification.

### Apparently convergent evolution of histone demethylases

The fact that the *KDM1 *and *JmjC *genes belong to two phylogenetically distinct gene families indicates that, during evolution, these two gene families were recruited to perform the histone demethylation activity independently, providing an example of convergent evolution. In fact, this phenomenon is prevalent among histone modifying enzymes. For instance, the enzymes that catalyze histone methylation belong to two different families, the widespread SET-domain family and the DOT1-related protein family [2]. Similarly, there are three distinct families of HDACs and four different families of HATs [63]. In addition, it is also common that families responsible for the same type of histone modification show distinct evolutionary patterns. While some families are widespread in eukaryotes (e.g. SET family HKMTs and SIR2 family HDACs), others are only present in specific lineages of eukaryotes (e.g. DOT1-related HKMTs in animals and fungi and HD2 family HDACs in plants) [2,63]. The recruitment of more than one gene families to fulfill the same type of biochemical activities might have allowed these families to evolve specific roles under different circumstances (e.g. cell type, developmental stage, environmental cues) or toward different substrates. The multiple origins of histone modification enzymes have likely contributed to the complexity of epigenetic regulation.

## Conclusion

In this paper, we present detailed phylogenetic analyses of the *KDM1 *and *JmjC *families, whose members include the recently identified histone demethylases. Our results revealed a possible single origin of all *KDM1 *histone demethylase genes through the acquisition of the region encoding the SWIRM domain by an *AOD *gene before the split of major eukaryotic lineages. The *KDM1 *genes are conserved in both copy number and domain structure during evolution, although a few duplication events were observed in plants. We also identified the contribution of HGT events to the evolution of *AOD *genes. On the other hand, our analyses *JmjC *genes showed this family clearly experienced birth-and-death evolution and the subfamilies displayed lineage-specific duplication patterns. According to the evolutionary relationship revealed by our study, we proposed histone demethylase activities for several plant JmjC domain-containing proteins. Furthermore, we found distinct evolutionary patterns of histone demethylases in different lineages and between the *KDM1 *and *JmjC *families. These results may imply functional divergence of certain types of histone methylation in different organisms and different classes of function associated with KDM1 and JmjC domain-containing histone demethylases. In summary, our study improves the understanding about the evolution and functions of histone demethylases and provides valuable information for future studies.

## Methods

### Data retrieval

The amino acid sequences of the AOD domain in reported KDM1 histone demethylases were retrieved from National Center for Biotechnology Information (NCBI). They were used as queries to search against NCBI, TAIR, TIGR and JGI databases for all possible AOD domain-containing proteins in selected eukaryotic organisms by using TBLASTN with e-value less than e^-5 ^as cut-off. All the new results were used as queries to carry out a second round of BLAST search, until no new sequence was found. The collected protein sequences were then analyzed by SMART and Pfam for domain architecture. The proteins which lack the AOD domain or have an AOD domain with e-value greater than e^-10 ^based on both SMART [68] and Pfam [69] results were excluded from the further analyses. The prokaryotic sequences were retrieved from NCBI database through BLASTP by using eukaryotic AOD domain-containing proteins as queries and e^-5 ^as cut-off. The same procedure was followed for the retrieval of JmjC domain-containing proteins. Common names for the following species are shown in the figures: Arabidopsis, *Arabidopsis thaliana*; Poplar, *Populus trichocarpa*; Rice, *Oryza sativa*; Moss, *Physcomitrella patens*; Human, *Homo sapiens*; Cow, *Bos taurus*; Mouse, *Mus musculus*; Zebrafish, *Danio rerio*; Fruitfly, *Drosophila melanogaster*; Mosquito, *Anopheles gambiae*; Honey bee, *Apis mellifera*; Beetle, *Tribolium castaneum*; Sea squirt, *Ciona intestinalis*; Sea urchin, *Strongylocentrotus purpuratus*; and Sea anemone, *Nematostella vectensis*.

### Sequence alignment

A preliminary multiple sequences alignment (MSA) was generated using MUSCLE 3.6 [70] with the default settings and a Neighbor-Joining (NJ) tree was constructed using MEGA 4.0 [71] based on the MSA. According to the tree topology, the sequences were divided into several subgroups. Each subgroup of sequences was aligned by MUSCLE 3.6 separately followed by manual adjustment using GeneDoc 2.6.0.3 [72]. These alignments were then combined using the profile alignment function of ClustalX 1.83 [73]. The codeml program from the PAML 4.1 package is used for the Ka/Ks analyses [74].

### Phylogenetic analysis

Both NJ and Maximum likelihood (ML) methods were used to perform the phylogenetic analyses. NJ trees were constructed using MEGA 4.0 with "pairwise deletion" option and "Poisson correction" model. Bootstrap test of 1000 replicates was carried out to evaluate the reliability of internal branches. ML trees were generated using PHYML 2.4.4 [75] with 100 nonparametric bootstrap replicates. ProtTest 1.4 [76] was used to select the model and parameters for the ML analysis. In this study, WAG amino acid substitution model was used and both proportion of invariable sites and gamma distribution parameter were estimated from the data. In this study, we presented only the NJ trees with bootstrap values from both NJ and ML analyses.

## Authors' contributions

XZ carried out the analysis and drafted the manuscript; HM conceived of and supervised the study, provided funding and critically revised the manuscript. All authors read and approved the final manuscript.

## Supplementary Material

Additional file 1 List of all the *AOD *genes and *JmjC *genes included in this study. Click here for file

Additional file 2 A NJ tree for fungal *KDM1 *genes. Click here for file

Additional file 3 Ka/Ks analysis of animal *KDM1A *and *KDM1B *genes. Click here for file
