# Supplementary figures and images for: Evolutionary history of histone demethylase families: distinct evolutionary patterns suggest functional divergence
Source: BMC Evol Biol. 2008 Oct 24;8:294. doi: 10.1186/1471-2148-8-294 (PMC2579438; doi:10.1186/1471-2148-8-294)

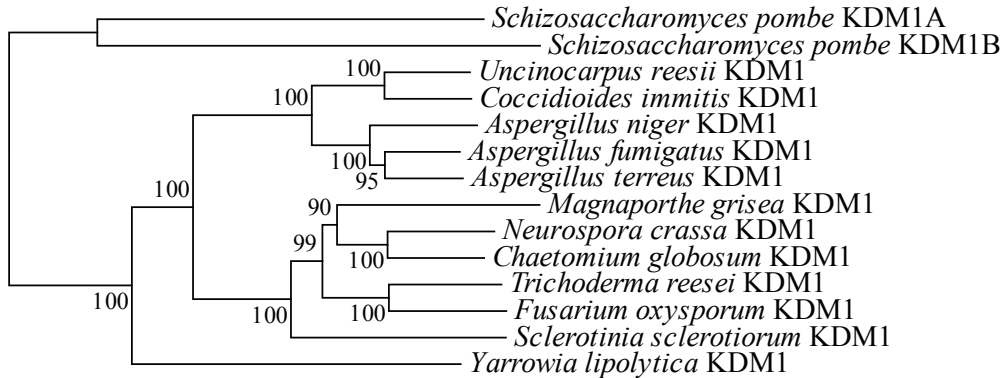

0.1

Supplement: Additional file 2 — A NJ tree for fungal KDM1 genes. [file 1471-2148-8-294-S2.pdf]
