# Supplementary material for: Evolutionary history of histone demethylase families: distinct evolutionary patterns suggest functional divergence
Source: BMC Evol Biol. 2008 Oct 24;8:294. doi: 10.1186/1471-2148-8-294 (PMC2579438; doi:10.1186/1471-2148-8-294)
Supplement: Additional file 3 — Ka/Ks analysis of animal KDM1A and KDM1B genes. [file 1471-2148-8-294-S3.pdf]

Supplementary Table 1: Ka/Ks analysis of animal *KDM1A* and *KDM1B* genes.

A. Ka/Ks ratios between animal *KDM1A* genes

	Cow	Mouse	Chicken	Frog
Human	0.0010	0.0010	0.0010	0.0036
	Pufferfish	Sea urchin	Sea anemone	Sea squirt
Human	0.0034	0.0052	0.0022	0.0080

B. Ka/Ks ratios between animal *KDM1B* genes

	Cow	Mouse	Chicken	Frog
Human	0.0945	0.0547	0.0613	0.0407
	Pufferfish	Sea urchin	Sea anemone	Sea squirt
Human	0.0256	0.0348	0.0289	0.0148

C. LRT for difference in Ka/Ks ratio between animal KDM1A and KDM1B clades

Models	$Ka/Ks_{(KDM1A)}$	$Ka/Ks_{(KDM1B)}$	Log-likelihood	LRT
One-ratio Model	0.0234	$= Ka/Ks_{(KDM1A)}$	-14180.1864	NA
Two-ratio Model	0.0036	0.0708	-14093.6031	$P < 0.0001$
